# Special Issue: Host Defense against Fungi

**DOI:** 10.3390/jof7121054

**Published:** 2021-12-09

**Authors:** Joseph M. Bliss

**Affiliations:** Department of Pediatrics, Women & Infants Hospital of Rhode Island, Warren Alpert Medical School of Brown University, Providence, RI 02905, USA; jbliss@wihri.org

Pathogenic fungi represent a small subset of a markedly diverse kingdom of organisms. They are characterized by biological adaptations that lead to colonization or infection of other “host” organisms—including plants, animals and humans—leading to some detriment in the host [1,2]. As such, host organisms have evolved multifaceted mechanisms to defend themselves in these interactions in ways that are as varied as the hosts themselves [3]. As the relevance of these infections increases due to factors such as environmental perturbations and alterations in human immune function through medical interventions, so has interest in better understanding these complex interactions. Not surprisingly, the contributions to this special issue are consistent with the conceptual and biological diversity of the field.

Although studies directed at understanding the mechanisms of fungal pathogenesis and defense mechanisms of the human host dominate the medical literature, the impact of pathogenic fungi on agriculture, the food chain, and global ecology should not be underestimated [4]. In this issue, Soni and colleagues present two studies addressing the issue of *Aspergillus flavus*- and *Aspergillus parasiticus*-produced aflatoxins as contaminants of maize and groundnut or peanut. These mycotoxins contaminate and accumulate in crops both before and after harvest as well as during storage and have been linked to hepatotoxic, carcinogenic, and teratogenic effects in humans [5]. Using RNA-seq to compare gene expression profiles from resistant and susceptible groundnut genotypes, the authors identify candidate genes that contribute to phenotypic resistance to aflatoxin production [6,7]. Additionally, study of nonmammalian hosts in the setting of fungal infections provides insight about the breadth of innate immune mechanisms employed. Rotskaya and colleagues describe the role of ricin-b-lectins in response to infection with *Metarhizium robertsii* and *Beauveria bassiana* in the Colorado potato beetle, *Leptinotarsa decemlineata* [8].

Pathogenic fungi have evolved powerful mechanisms to evade, subvert, or alter host immunity to their benefit. This issue provides a review by Mendoza and colleagues of fungal and host-derived eicosanoids that have immunoregulatory properties and may serve as a potential target in therapeutic strategies [9]. Williams and colleagues review how induction of a variety of programmed cell death pathways can be elicited by pathogenic fungi to potentially gain an advantage in the infectious process [10]. In an article highlighting the complexity of these interactions, Montoya and colleagues review how varied genotypes and phenotypes in *Cryptococcus* are associated with human disease and, conversely, how variability among human clinical phenotypes impacts disease outcomes [11].

Inquiries into the host side of the host–fungus interaction continue to demonstrate novel mechanisms of host defense and susceptibility, with advances both in discovery and methodology. The contribution of Cicuéndez and colleagues to this issue describes insights gained using a decellularized adipose matrix to evaluate the influence of key components of the extracellular environment on the interaction between macrophages and *Candida albicans*, an angle often lacking in studies in vitro [12]. The influence of the microenvironment on macrophage–fungi interactions is also featured in a study by Luvanda and colleagues, in which the effect of dexamethasone on *Aspergillus fumigatus* is investigated in a physiologically relevant air–liquid interface epithelial/immune lung model [13]. The contribution of degranulation by a variety of immune cells in defense against fungi has been reviewed by Mok and colleagues [14]. In novel work conducted by dos Santos Dias et al., the critical role of neutrophils in an effective immune response to a therapeutic vaccine, P10 + DODAB, is demonstrated in a murine model of Paracoccidioidomycosis [15]. Finally, Adrizzoni et al. have provided data supporting a role for perinuclear anti-neutrophil cytoplasmic antibodies (pANCA) in vaginal fluid from women with symptomatic vulvovaginal candidiasis that may impair neutrophil function against *Candida* in this environment [16].

This issue also features several articles of interest to clinicians who manage patients with the many manifestations of fungal disease. An overview of fungal keratitis in the United Kingdom through the most recent decade is provided by Ting et al. [17], and oral candidiasis is the topic of a comprehensive review by Lu [18]. Celakovska and colleagues have reported on the relationship between sensitization to molecular components of environmental fungi and severity of atopic dermatitis in a cohort of 100 patients [19]. A comprehensive review of *Cryptococcus gatii* infections in patients with lymphoid neoplasms including relevant aspects of host–pathogen interactions has been provided by Paccoud et al. [20]. 

Prompt and accurate diagnosis of invasive fungal disease remains a challenge in the clinic, and expanding on existing treatment strategies remains of critical importance in vulnerable populations. In their study, Vaz et al. purified and analyzed the *C. albicans* hyphal secretome by liquid chromatography–tandem mass spectrometry and compared the immunoreactivity patterns of human serum samples from patients with and without invasive candidiasis. Antibody titers to several secreted proteins enabled discrimination between infected and uninfected patients and were proposed as potential diagnostic biomarkers for further study [21]. A review by Ankrah at al. provides an overview of radionuclide imaging as an adjunct for diagnosis, as well as staging of dissemination and response to treatment in fungal infections, and includes a discussion of tracers at the preclinical stage [22]. Finally, a review of strategies to augment host defense through immunomodulatory approaches has been provided by Karavalakis and colleagues, with a focus on T-cell immunotherapy against invasive fungal disease in severely immunocompromised patients [23].

I very much appreciate the contributions of each of the authors and thank them for sharing their expertise in this Special Issue on Host Defense against Fungi.

## References

[1] Kohler J.R., Hube B., Puccia R., Casadevall A., Perfect J.R. (2017). Fungi that Infect Humans. Microbiol. Spectr..

[2] Lo Presti L., Lanver D., Schweizer G., Tanaka S., Liang L., Tollot M., Zuccaro A., Reissmann S., Kahmann R. (2015). Fungal effectors and plant susceptibility. Annu. Rev. Plant Biol..

[3] Dambuza I.M., Levitz S.M., Netea M.G., Brown G.D. (2017). Fungal Recognition and Host Defense Mechanisms. Microbiol. Spectr..

[4] Fisher M.C., Gurr S.J., Cuomo C.A., Blehert D.S., Jin H., Stukenbrock E.H., Stajich J.E., Kahmann R., Boone C., Denning D.W. (2020). Threats Posed by the Fungal Kingdom to Humans, Wildlife, and Agriculture. mBio.

[5] Kumar P., Mahato D.K., Kamle M., Mohanta T.K., Kang S.G. (2016). Aflatoxins: A Global Concern for Food Safety, Human Health and Their Management. Front. Microbiol..

[6] Soni P., Pandey A.K., Nayak S.N., Pandey M.K., Tolani P., Pandey S., Sudini H.K., Bajaj P., Fountain J.C., Singam P. (2021). Global Transcriptome Profiling Identified Transcription Factors, Biological Process, and Associated Pathways for Pre-Harvest Aflatoxin Contamination in Groundnut. J. Fungi.

[7] Soni P., Nayak S.N., Kumar R., Pandey M.K., Singh N., Sudini H.K., Bajaj P., Fountain J.C., Singam P., Hong Y. (2020). Transcriptome Analysis Identified Coordinated Control of Key Pathways Regulating Cellular Physiology and Metabolism upon *Aspergillus flavus* Infection Resulting in Reduced Aflatoxin Production in Groundnut. J. Fungi.

[8] Rotskaya U.N., Kryukov V.Y., Kosman E., Tyurin M., Glupov V.V. (2021). Identification of the Ricin-B-Lectin LdRBLk in the Colorado Potato Beetle and an Analysis of Its Expression in Response to Fungal Infections. J. Fungi.

[9] Mendoza S.R., Zamith-Miranda D., Takacs T., Gacser A., Nosanchuk J.D., Guimaraes A.J. (2021). Complex and Controversial Roles of Eicosanoids in Fungal Pathogenesis. J. Fungi.

[10] Williams T.J., Gonzales-Huerta L.E., Armstrong-James D. (2021). Fungal-Induced Programmed Cell Death. J. Fungi.

[11] Montoya M.C., Magwene P.M., Perfect J.R. (2021). Associations between *Cryptococcus* Genotypes, Phenotypes, and Clinical Parameters of Human Disease: A Review. J. Fungi.

[12] Cicuéndez M., Casarrubios L., Feito M.J., Madarieta I., Garcia-Urkia N., Murua O., Olalde B., Briz N., Diez-Orejas R., Portoles M.T. (2021). *Candida albicans*/Macrophage Biointerface on Human and Porcine Decellularized Adipose Matrices. J. Fungi.

[13] Luvanda M.K., Posch W., Noureen A., Lafon E., Zaderer V., Lass-Florl C., Wilflingseder D. (2021). Dexamethasone Creates a Suppressive Microenvironment and Promotes *Aspergillus fumigatus* Invasion in a Human 3D Epithelial/Immune Respiratory Model. J. Fungi.

[14] Mok A.C., Mody C.H., Li S.S. (2021). Immune Cell Degranulation in Fungal Host Defence. J. Fungi.

[15] Dias L.D.S., Silva L.B.R., Nosanchuk J.D., Taborda C.P. (2021). Neutrophil Cells Are Essential for The Efficacy of a Therapeutic Vaccine against Paracoccidioidomycosis. J. Fungi.

[16] Ardizzoni A., Sala A., Colombari B., Giva L.B., Cermelli C., Peppoloni S., Vecchiarelli A., Roselletti E., Blasi E., Wheeler R.T. (2020). Perinuclear Anti-Neutrophil Cytoplasmic Antibodies (pANCA) Impair Neutrophil Candidacidal Activity and Are Increased in the Cellular Fraction of Vaginal Samples from Women with Vulvovaginal Candidiasis. J. Fungi.

[17] Ting D.S.J., Galal M., Kulkarni B., Elalfy M.S., Lake D., Hamada S., Said D.G., Dua H.S. (2021). Clinical Characteristics and Outcomes of Fungal Keratitis in the United Kingdom 2011–2020: A 10-Year Study. J. Fungi.

[18] Lu S.Y. (2021). Oral Candidosis: Pathophysiology and Best Practice for Diagnosis, Classification, and Successful Management. J. Fungi.

[19] Celakovska J., Vankova R., Bukac J., Cermakova E., Andrys C., Krejsek J. (2021). Atopic Dermatitis and Sensitisation to Molecular Components of *Alternaria*, *Cladosporium*, *Penicillium*, *Aspergillus*, and *Malassezia*-Results of Allergy Explorer ALEX 2. J. Fungi.

[20] Paccoud O., Bougnoux M.E., Desnos-Ollivier M., Varet B., Lortholary O., Lanternier F. (2021). *Cryptococcus gattii* in Patients with Lymphoid Neoplasms: An Illustration of Evolutive Host-Fungus Interactions. J. Fungi.

[21] Vaz C., Pitarch A., Gomez-Molero E., Amador-Garcia A., Weig M., Bader O., Monteoliva L., Gil C. (2021). Mass Spectrometry-Based Proteomic and Immunoproteomic Analyses of the *Candida albicans* Hyphal Secretome Reveal Diagnostic Biomarker Candidates for Invasive Candidiasis. J. Fungi.

[22] Ankrah A.O., Sathekge M.M., Dierckx R., Glaudemans A. (2021). Radionuclide Imaging of Fungal Infections and Correlation with the Host Defense Response. J. Fungi.

[23] Karavalakis G., Yannaki E., Papadopoulou A. (2021). Reinforcing the Immunocompromised Host Defense against Fungi: Progress beyond the Current State of the Art. J. Fungi.

